# Development of a registry to evaluate immobilized lipase cartridge use in pediatric patients with short bowel syndrome/intestinal failure^☆^

**DOI:** 10.1016/j.intf.2025.100036

**Published:** 2025-01-16

**Authors:** Jason Soden, Megan Aarnio-Peterson, Justin Neal, David P. Recker, Ann E. Remmers

**Affiliations:** a Section of Pediatric Gastroenterology, Hepatology, and Nutrition: University of Colorado School of Medicine and Children’s Hospital Colorado, 13123 E 16th Avenue, Aurora, CO 80045, United States; b Alcresta Therapeutics, 130 Turner Street, Building 3, Suite 200, Waltham, MA 02453, United States; c PRO-spectus, LLC, 412 Olive Ave., Ste 271, Huntington Beach, CA 92648, United States

**Keywords:** Intestinal failure, Short bowel syndrome, Fat malabsorption, Immobilized lipase cartridge, Enteral nutrition, Registry

## Abstract

**Background:**

Over the past 4 years, outpatient use of an immobilized lipase cartridge (ILC) (RELiZORB®; Alcresta Therapeutics) in pediatric patients with intestinal failure (IF) and short bowel syndrome (SBS) has increased. Although ILC use has been associated with improved fat absorption and enteral feeding tolerance in patients with exocrine pancreatic insufficiency and cystic fibrosis, there are no published reports of ILC use in patients with IF.

**Materials and methods:**

This prospective direct-to-patient observational registry collects real-world data regarding medical history, growth, quality of life, and progression towards enteral autonomy in pediatric patients with IF/SBS receiving enteral nutrition administered through an ILC. Eligible patients identified by a registry clinical coordinator are given the option to enroll in the registry. Data collected for the registry includes caregiver-as-proxy patient-reported outcome questionnaires, and clinical data from routine clinic visits for each patient for up to one year. A Registry Steering Committee provides oversight and reviews the scientific merit of proposals for subsequent data analysis.

**Results:**

The registry received Institutional Review Board approval in July 2024 with enrollment commencing shortly thereafter. As of October 11, 2024, 41 patients receiving care at 25 institutions in the United States were enrolled in the registry.

**Conclusion:**

It is anticipated that data collected through this registry will allow study of relevant research questions related to ILC use in patients with IF/SBS, including queries related to growth, progression towards enteral autonomy, and quality of life.

## Highlights

• A direct-to-patient registry is collecting real-world data in pediatric patients with SBS/IF.
• Patients receiving enteral nutrition through an immobilized lipase cartridge are eligible.
• This registry offers a practical solution to data collection in this rare disease.

## Introduction

Patients with intestinal failure (IF) and short bowel syndrome (SBS) experience fluid, electrolyte, and nutrient malabsorption. The clinical manifestations of malabsorption are heterogeneous, due to the variety of congenital and acquired anatomic abnormalities that might lead to SBS. Because of various pathophysiologic alterations, fat malabsorption is an important factor in many patients with SBS. Anatomic alterations may affect fat digestion and absorption through decreased jejunal absorptive surface area, rapid transit, impact on ileal bile acid reabsorption, small intestinal bacterial overgrowth, and other factors [1]. Clinical manifestations of fat malabsorption may include calorie malabsorption / malnutrition, alterations in fat-soluble vitamin status, and alterations in essential fatty acid status.

Fat malabsorption in SBS patients was observed in a cohort study of 90 adults with SBS and a functional pancreas, in whom the mean coefficient of fat absorption ranged from 47 % to 61 % [2]. Additionally, in a small phase 2 study, a subset of SBS patients without exocrine pancreatic insufficiency demonstrated improved fat absorption with pancreatic enzyme treatment [3]. These observations suggest that there may be a role for exogenous lipase supplementation to improve luminal fat digestion and absorption in some patients with SBS.

RELiZORB (Alcresta Therapeutics, Waltham, MA) is a single-use, point-of-care immobilized lipase cartridge (ILC) that connects in-line with existing enteral feeding supplies and is designed to hydrolyze triglycerides in enteral formulas, allowing for the delivery of absorbable fatty acids and monoglycerides to patients who rely on tube feeding. Although ILC use has been associated with improved fat absorption and enteral feeding tolerance in patients with exocrine pancreatic insufficiency and cystic fibrosis, there are no published reports of ILC use in patients with IF or SBS [4], [5].

Results from two preclinical studies evaluating ILC use in a porcine model of SBS with IF have been reported. In the first study, ILC use with enteral feeds in piglets with a 75 % jejunal resection was associated with improved fat and fat-soluble vitamin absorption compared to resected controls [6]. ILC usage was not associated with an increased frequency of adverse events compared to the other groups. The second study demonstrated that ILC use in conjunction with enteral feeding reduced PN dependence, improved nutrient absorption, and increased bowel growth [7]. These studies demonstrate that ILC use with EN improves nutrient and fat absorption in clinical models of IF with SBS while promoting enteral autonomy (EA) through reduction of PN dependence.

In recent years, outpatient use of ILC in patients with IF/SBS has been increasing (manufacturer internal records), although no studies evaluating ILC usage in patients with IF/SBS have been published. A prospective single-center, single-arm clinical study evaluating ILC use is ongoing in pediatric patients with SBS who are PN dependent (NCT03530852) [8]. This study hypothesizes that ILC usage will enhance EA through increased fat absorption via EN, leading to reduced dependence on PN. No other studies of interventions to treat fat malabsorption in SBS are ongoing. Although an ongoing International Intestinal Failure Registry assesses the natural history of IF in pediatric patients and identifies evidence-based interventions, the role of either pancreatic enzyme supplementation or formula modification is currently not being evaluated in that registry [9].

A prospective direct-to-patient observational registry has been created to collect real-world data including medical history, growth, quality of life, and progression towards EA in pediatric patients with IF/SBS receiving enteral nutrition (EN) administered through an ILC. The planning and implementation of this registry follows applicable guidelines prepared for the Agency for Healthcare Research and Quality [10] as well as methodology standards from the Patient-Centered Outcomes Research Institute [11]. The registry, which is sponsored and funded by the ILC manufacturer, is designed to support the development of treatment guidelines regarding the use of an ILC in patients with IF/SBS.

## Methods

### Study design

In this prospective direct-to-patient registry, a third party contracted by the sponsor recruits patients and collects data, by direct interaction with patients’ caregivers [12]. Data are also provided by the treating healthcare providers. Study design, data collection, and data storage are performed in compliance with relevant laws and institutional guidelines and have been approved by the appropriate institutional committee(s).

### Registry administrative structure and oversight

The sponsor has an existing contract with the third-party vendor Pro-spectus, LLC (Huntington Beach, CA) to administer RELiZORB Support Services (RSS), which provides ongoing reimbursement support and insurance authorization for ILC usage through receipt of outpatient prescriptions. The sponsor contracted with Pro-spectus to develop a separate registry database of pediatric patients with IF/SBS receiving EN through an ILC. The patient support program is independent from the registry clinical team, such that participation in the registry is not a prerequisite for ILC prescription fulfillment. Central IRB approval was granted for RSS to serve as the single investigator and clinical site to identify eligible patients, obtain consent, and collect patient clinical data and caregiver-as-proxy patient-reported outcomes. RSS also created and maintains a 21 CFR Part 11 validated data repository to store caregiver-as-proxy patient-reported outcome responses to questionnaires.

The third-party vendor ClaritasRx (San Francisco, CA) developed and maintains the RSS reimbursement database where patient clinical records are stored. The sponsor contracted with ClaritasRx to create a separate database to capture registry-specific data fields for patients enrolled in the registry. The Claritas registry database complies with 21 CFR Part 11 regulations and is pending software validation.

A Steering Committee consisting of a Coordinating Investigator (Committee Chair), Principal Investigator, and sponsor Medical Monitor provides oversight for the registry. The committee provided input on the information collected in the registry and will review the scientific merit of proposals for subsequent analysis of registry data. The committee will convene periodically to ensure that data quality is adequate for future analyses. Duration and maximum enrollment of the registry are not set; those questions will be decided by the Steering Committee. It is anticipated that approximately 200 patients from more than 100 centers in the United States (US) will enroll in the registry in the first year.

### Patient eligibility criteria and rationale

Pediatric patients (21 years or younger) must meet the following criteria: receiving or plan to receive EN as part of their ongoing care for treatment of GI or digestive conditions; PN use > 60 days (ongoing or historic use) due to intestinal disease, dysfunction, or resection; initiation of ILC use after May 13, 2024; attendance at a minimum of quarterly patient clinic follow-up visits; and Health Insurance Portability and Accountability Act (HIPAA) consent and patient authorization signed by the patient’s legal guardian. Patients with a diagnosis of cystic fibrosis and exocrine pancreatic insufficiency with no additional comorbid GI conditions are excluded from the registry.

The patient population to be included in the registry is in line with the definition of IF established by the Nutrition Committee of the North American Society for Pediatric Gastroenterology, Hepatology, and Nutrition [13]. The patient’s HIPAA authorization is required by all patients prescribed an ILC, regardless of registry participation. The ILC prescription enrollment form and signed patient/caregiver authorization form (https://www.relizorb.com/support-and-resources/) are necessary for RSS to obtain patient medical records in support of insurance authorization. The patient/caregiver authorization form allows RSS to collect patient information related to ILC treatment, safety, and effectiveness. The registry clinical coordinator reviews medical records for registry eligibility and contacts eligible patients’ caregivers to educate them about the registry. An additional consent is required for registry enrollment. Because registry participation presents no more than minimal risk of harm to subjects and involves no procedures for which written consent is normally required outside of the research context (45 CFR 46.117(c)(1)(ii)), the central IRB approved a waiver of written consent. Verbal parental consent is obtained for patients younger than the age of majority; verbal patient assent is also obtained for patients 7–17 years of age. For children incapable of providing assent, only parental verbal consent is collected. To minimize caregiver burden, caregivers have the option to opt out of questionnaire completion during informed consent yet still participate in the registry.

### Data collection

Following consent, the registry clinical coordinator notifies the prescribing healthcare provider of the patient’s enrollment in the registry. RSS will collect data from healthcare providers periodically following regularly scheduled follow-up visits, at approximately 3-, 6-, 9-, and 12-months post initiation of ILC. Following receipt of data via fax or secure email from healthcare providers, RSS will record data from clinic notes into the database. Data collected at baseline and following initiation of ILC use are listed in Table 1. The reason for discontinuation of ILC will be collected for patients who discontinue use before 12 months. Missing data are likely for some patients and will be taken into account in any subsequent data analysis.

**Table 1:** Registry data collection.

**Evaluation**	**Data Variables**
**Baseline**	
Demographics and baseline characteristics	Unique patient ID, Referring Hospital Name, Patient date of birth, Sex, height, weight, BMI, current PN/EN use
Patient-Reported Outcomes	Global Health Peds QL [14] and GI symptom questionnaires [15] (caregiver-as-proxy for patient)
Medical History	Primary diagnosis for referral, Primary/Secondary/Tertiary ICD 10 codes, exocrine pancreatic insufficiency diagnosis (yes/no), premature birth, significant cardiopulmonary disease, seizure disorder, developmental delay, feeding difficulties
Surgical History	Type of bowel resection and date, length of small intestine remaining, type of stoma, ileocecal valve present, intestinal transplantation date
**Follow-up (collected from clinic notes at periodic clinic/nutrition visits and hospitalizations for up to 1 year ( ± 2 months))**	
Anthropometrics	Height, Weight, BMI
Parenteral Nutrition Use	Total daily calories, hours/day
Enteral Formula use	Name of enteral formula, total daily calories, hours/day
Patient-Reported Outcomes	Global Health PedsQL and GI symptom questionnaires at 1 and 12 months following ILC initiation Caregiver assessment of change at 1 and 12 months following ILC initiation
Clinical Laboratory Assessments	Fat soluble vitamins (for patients who initiate ILC use when receiving enteral nutrition only – no PN)
Concomitant medication	Teduglutide (Gattex) use and start/stop dates
Hospitalization	Dates of hospitalization and reason
ILC use	Start Date / First Ship Date, Last Ship Date, ongoing at 1 year, reason for discontinuation, Number of cartridges prescribed

No registry-specific visits or procedures conducted by the healthcare provider are considered to be research by the central IRB. Therefore, local IRB review and approval at each participating healthcare provider’s institution is not required by the central IRB. Should local IRB approval be necessary per institutional standards, the sponsor will work directly with the prescribing healthcare provider’s site to facilitate any site-specific IRB submissions. The sponsor will work directly with clinics to put a contract in place to cover periodic provision of medical records including physician and dietician notes. The contract includes financial support for work performed in support of the registry.

RSS will contact caregivers to obtain caregiver-as-proxy patient-reported outcomes at baseline, 1 and 12 months following ILC initiation (Table 2). Questionnaires are completed electronically or by telephone according to the caregiver’s preference. Caregivers will receive nominal compensation following their completion of the final questionnaires.

**Table 2:** Collection of Caregiver-as-Proxy Patient-Reported Outcomes.

**Caregiver completes questionnaires**	**Baseline** **(prior to ILC use)**	**Month 1** **( ± 1 week)**	**Month 12** **( ± 2 months)**
PedsQL	X	X	X
PedsQL GI Symptoms	X	X	X
Caregiver assessment of change		X	X

### Patient-reported outcomes

The Pediatric Quality of Life Inventory (PedsQL) and PedsQL Gastrointestinal (GI) Symptoms are validated health-related quality-of-life questionnaires that are completed by caregivers [14, 15; licensed for use by Mapi Research Trust, Lyon, France, https://eprovide.mapi-trust.org]. The PedsQL is a modular instrument for measuring health-related quality of life (HRQOL) in children, adolescents, and young adults ages 2–25 [14]. The PedsQL captures physical health, psychosocial health, emotional functioning, social functioning, and school functioning. The caregiver-as-proxy report includes age-specific questionnaires for ages 2–4 (toddler), 5–7 (young child), 8–12 (child), 13–18 (adolescent), and 19–21 years of age (young adult) to assess each caregiver’s perceptions of their child’s HRQOL.

The PedsQL GI Symptoms module is specific for patients with functional GI disorders and organic GI diseases. Each of the 14 symptom scales was designed to be a standalone symptom scale [16]. To minimize caregiver burden, the 7-item diarrhea scale is initially being used in this registry; additional symptom scales may be used in the future. In addition, the Caregiver Assessment of Change questionnaire will be completed 1 month and 12 months after ILC initiation to assess how the patient’s GI problems have changed. The Caregiver Assessment of Change is a standard 7-point research measurement to assess global change in health.

### Data quality

To ensure data quality, source data verification (SDV) of patient medical records will be completed by a second registry team member trained by the registry clinical coordinator. SDV is performed for all height, weight, and PN calorie measurements for each registry participant. Claritas registry database records will be reviewed on an ongoing basis and any discrepancies in data will be corrected. Prior to each database snapshot in preparation for a Steering Committee meeting, verification of existing height, weight, and PN information will be completed as well as requests for any outstanding medical records. To ensure that eligible patients are enrolled in the registry, RSS will consult with the Coordinating Investigator.

## Results

The registry protocol received WCG central IRB approval on July 18, 2024 with enrollment of the first patient shortly thereafter. As of October 11, 2024, 41 patients receiving care at 25 institutions in the US were enrolled in the registry. The median age at ILC initiation in this initial group of patients is 7 years; a majority (63 %) of the patients are between the ages of 3 and 12 (Table 3). The most common reason for IF is SBS; necrotizing enterocolitis is the cause of SBS in 49 % of these patients. Although all patients had a history of PN use, 24 % of patients are no longer receiving PN and were only receiving EN at the time of initiation of ILC use.

**Table 3:** Baseline patient demographics and baseline characteristics.

Variable/Characteristic	Enrolled Patients (N = 41)
Age, years, mean (SD) Range (min, max)	7.0 (4.6) 0, 19
Age group, years, n (%)	
0 – 2 3 – 12 13 – 19	9 (22 %) 26 (63 %) 6 (15 %)
Sex, n (%)	
Male	24 (59 %)
SBS Diagnosis, n (%)	35 (85 %)
Reason for SBS, n (%)	
Necrotizing enterocolitis Midgut volvulus Intestinal atresia Gastroschisis Multiple Reason pending	17 (49 %) 6 (17 %) 5 (14 %) 4 (11 %) 2 (6 %) 1 (3 %)
Supplemental Nutrition, n (%)	
Parenteral and Enteral Enteral	31 (76 %) 10 (24 %)

## Discussion

Registries play a particularly important role in the study of rare diseases. The small number of affected patients within individual healthcare centers makes clinical center-based registry enrollment challenging. Direct-to-patient registries have been shown to be effective because they allow for patient enrollment from smaller/regional institutions that may not have the resources to participate in clinical studies [12]. This registry is unique as it is managed by a third-party patient support program. In clinical practice, prescribing providers have become increasingly accustomed to sending prescriptions to patient support programs, which offer ease of use and a greater rate of prescription approval/fulfillment than typical pharmacy services. This registry seeks to leverage that clinical trend, while giving patients and providers the opportunity to participate in research on one treatment option for a rare disease.

Once patients have enrolled and consented to registry participation, the registry operates alongside the patient support program, acting as an intermediary to which prescribers routinely provide clinical information as part of the traditional approval process. Once the patient consents to registry participation, the questionnaires are administered to the caregivers by a familiar entity that has been in communication with each caregiver regarding ILC use. As self-directed registries have already been hypothesized to have lower discontinuation rates [12], patient support programs that are already engaged in communicating with caregivers/patients will likely mitigate patient loss during the 12-month follow-up period.

We anticipate that future research proposals based on the registry’s real-world data will be able to address several important questions related to patient growth, quality of life, and progression towards EA in patients with IF/SBS, in particular regarding the utility of ILC in this population of patients. The endpoints PN use, growth, and HRQOL are in the core pediatric chronic intestinal failure outcome set and are in alignment with previously published key outcome data points [17]. Patient information regarding growth and use of supplemental PN and EN are routinely collected and available in clinic notes in consistent units (e.g., kg for weight, cm for height, kcal/day for PN and EN) across US sites. Medical records also provide fairly standardized reports of each patient’s medical and surgical history, so variables relevant to time to achieve EA [18] are being collected in the registry.

In addition to these well-defined nutritional outcomes, this registry collects information regarding patient quality of life; however, use of the caregiver-as-proxy may introduce bias. Elevated stress and anxiety and decreased HRQOL were reported in an exploratory survey of caregivers of children with IF receiving PN [19]. Caregiver burden or other factors may negatively impact a caregiver’s responses regarding the child’s QOL, as was reported in an exploratory study of parent and child HRQOL for children with SBS [20]. To our knowledge, this is the only registry incorporating PedsQL and PedsQL GI questionnaires for this IF patient population. Few quality of life and gastrointestinal symptoms questionnaires have been validated for pediatric patients. Given the wide range of patients eligible for the registry and the anticipated significant enrollment of children less than 5 years of age, for whom no patient-directed version of the questionnaires has been validated, the caregiver-as-proxy versions of the questionnaires for patients 2–25 years of age will be used in the registry.

Limitations for this type of registry include the risk of incomplete data due to caregiver lack of response to questionnaires or incomplete collection of medical records. In addition, the variety of additional treatments in the clinical management of IF/SBS (e.g., formulas, medications, surgeries) may confound outcomes, if other changes in treatment are concurrent with ILC initiation.

## Conclusion

More data are needed to help guide clinicians about treatment strategies to improve EN absorption, improve symptoms, and decrease PN burden in patients with IF/SBS. The design of the registry offers a practical solution to the problem of collecting data in this rare and heterogeneous disorder: partnership directly between caregivers/patients and a third-party patient support program that is already engaged in ILC prescription fulfillment for tube-fed SBS patients. In addition, patients and caregivers may appreciate the opportunity to help advance knowledge in IF/SBS. Furthermore, clinicians and care teams may have increased access to participate in the registry based on pre-established central IRB approval. It is anticipated that future research based on registry real-world data will be able to address multiple questions related to the role of ILC use in IF/SBS, and the potential impact of this novel therapy on growth, quality of life, and progression towards EA in patients with IF/SBS.

## Funding

This study is funded by Alcresta Therapeutics, Inc.

## Ethical statement

Study design, data collection, and data storage are performed in compliance with relevant laws and institutional guidelines and have been approved by the appropriate institutional committee(s). The registry protocol received WCG central IRB approval on July 18, 2024 (IRB tracking number 20242872).

## Patient's/Guardian's consent

Patients consented to publishing the data with the knowledge that their identity would not be disclosed.

## CRediT authorship contribution statement

**Jason Soden:** Writing – review & editing, Writing – original draft, Methodology, Data curation. **Megan Aarnio-Peterson:** Writing – review & editing, Writing – original draft, Validation, Supervision, Project administration, Methodology, Investigation, Data curation. **Justin Neal:** Writing – review & editing, Validation, Project administration, Investigation. **David P. Recker:** Writing – review & editing, Supervision, Resources, Methodology, Conceptualization. **Ann E. Remmers:** Writing – review & editing, Writing – original draft, Supervision, Methodology, Investigation, Conceptualization.

## Declaration of Competing Interest

The authors declare the following financial interests/personal relationships which may be considered as potential competing interests: David Recker reports a relationship with Alcresta Therapeutics Inc that includes: consulting or advisory. Megan Aarnio-Peterson reports a relationship with Alcresta Therapeutics Inc that includes: employment. Jason Soden reports a relationship with Alcresta Therapeutics Inc that includes: consulting or advisory and travel reimbursement. Justin Neal reports a relationship with Alcresta Therapeutics Inc that includes: funding grants. Ann E Remmers reports a relationship with Alcresta Therapeutics Inc that includes: employment. If there are other authors, they declare that they have no known competing financial interests or personal relationships that could have appeared to influence the work reported in this paper.
